# Multi-Organ Histopathological Changes in a Mouse Hepatitis Virus Model of COVID-19

**DOI:** 10.3390/v13091703

**Published:** 2021-08-27

**Authors:** Michael J. Paidas, Adhar B. Mohamed, Michael D. Norenberg, Ali Saad, Ariel Faye Barry, Cristina Colon, Norma Sue Kenyon, Arumugam R. Jayakumar

**Affiliations:** 1 Departments of Obstetrics, Gynecology and Reproductive Sciences, University of Miami, Miami, FL 33136, USA; adi.mohamed@med.miami.edu (A.B.M.); a.barry1@med.miami.edu (A.F.B.); cbc97@miami.edu (C.C.); 2 Division of Neuropathology, Department of Pathology and Laboratory Medicine, University of Miami Miller School of Medicine, Miami, FL 33136, USA; mnorenbe@med.miami.edu (M.D.N.); axs3162@med.miami.edu (A.S.); 3 Microbiology & Immunology and Biomedical Engineering, Diabetes Research Institute, University of Miami, Miami, FL 33136, USA; NKenyon@med.miami.edu

**Keywords:** COVID-19, SARS-CoV-2, mice, multiorgan histopathology, mouse hepatitis virus-1, vascular defect

## Abstract

Infection with SARS-CoV-2, the virus responsible for the global COVID-19 pandemic, causes a respiratory illness that can severely impact other organ systems and is possibly precipitated by cytokine storm, septic shock, thrombosis, and oxidative stress. SARS-CoV-2 infected individuals may be asymptomatic or may experience mild, moderate, or severe symptoms with or without pneumonia. The mechanisms by which SARS-CoV-2 infects humans are largely unknown. Mouse hepatitis virus 1 (MHV-1)-induced infection was used as a highly relevant surrogate animal model for this study. We further characterized this animal model and compared it with SARS-CoV-2 infection in humans. MHV-1 inoculated mice displayed death as well as weight loss, as reported earlier. We showed that MHV-1-infected mice at days 7–8 exhibit severe lung inflammation, peribronchiolar interstitial infiltration, bronchiolar epithelial cell necrosis and intra-alveolar necrotic debris, alveolar exudation (surrounding alveolar walls have capillaries that are dilated and filled with red blood cells), mononuclear cell infiltration, hyaline membrane formation, the presence of hemosiderin-laden macrophages, and interstitial edema. When compared to uninfected mice, the infected mice showed severe liver vascular congestion, luminal thrombosis of portal and sinusoidal vessels, hepatocyte degeneration, cell necrosis, and hemorrhagic changes. Proximal and distal tubular necrosis, hemorrhage in interstitial tissue, and the vacuolation of renal tubules were observed. The heart showed severe interstitial edema, vascular congestion, and dilation, as well as red blood cell extravasation into the interstitium. Upon examination of the MHV-1 infected mice brain, we observed congested blood vessels, perivascular cavitation, cortical pericellular halos, vacuolation of neuropils, darkly stained nuclei, pyknotic nuclei, and associated vacuolation of the neuropil in the cortex, as well as acute eosinophilic necrosis and necrotic neurons with fragmented nuclei and vacuolation in the hippocampus. Our findings suggest that the widespread thrombotic events observed in the surrogate animal model for SARS-CoV-2 mimic the reported findings in SARS-CoV-2 infected humans, representing a highly relevant and safe animal model for the study of the pathophysiologic mechanisms of SARS-CoV-2 for potential therapeutic interventions.

## 1. Introduction

Infection with SARS-CoV-2, the virus responsible for the global COVID-19 pandemic, primarily causes a respiratory illness. Approximately 193 million cases have been reported worldwide, resulting in more than 4 million deaths to date. COVID-19 is likely precipitated by cytokine storm, septic shock, thrombosis, and oxidative stress [1]. Infected individuals develop a wide range of symptoms ranging from mild to severe illness. Symptoms can appear 2–14 days after exposure to the virus. These include, fever, chills, loss of taste or smell, sore throat and cough, shortness of breath or difficulty in breathing, congestion and runny nose, fatigue, muscle and body aches, severe headache, nausea or vomiting, and diarrhea [2,3].

Most individuals (81%) develop mild to moderate symptoms of COVID-19, which can extend to pneumonia [4,5,6,7,8,9,10,11]. However, 14% of patients develop severe symptoms that include dyspnea and hypoxia, with more than 50% showing lung involvement [12,13]. Additionally, 5% of patients suffer critical symptoms such as respiratory failure and shock. At least one third of individuals infected with SARS-CoV-2 virus do not develop noticeable symptoms at any point in time [14]. Asymptomatic carriers tend to not get tested, thus spreading the virus to others and leading to an increase in infections. Individuals may develop symptoms later (pre-symptomatic) or have very mild symptoms [14]. Recently, multiple SARS-CoV-2 variants were reported to be circulating globally. These include a UK variant (B.1.1.7, also known as VOC-202012/01), South African variants (B.1.351, 501Y.V2), and Brazilian variants of SARS-CoV-2 (P.1, B.1.1.28.1, and B.1.617.2). These variants have been classified by the CDC as variants of concern [15] due to their high rate of transmissibility, although their epidemiology and impact on humans are unclear. More recently, the additional SARS-CoV-2 variants B.1.427, B.1.429, B.1.525, P.3, B.1.617.1, B.1.620, B.1.621, and C.37 (variants of interest), as well as B.1.617.3, B.1.214.2, A.23.1+E484K, A.27, A.28, C.16, B.1.351 + P384L, B.1.351 + E516Q, B.1.1.7 + L452R, B.1.1.7 + S494P, C.36 + L452R, AT.1, B.1.526, B.1.526.1, B.1.526.2, B.1.1.318, P.2, B.1.1.519, AV.1, P.1 + P681H, B.1.671.2 + K417N (variants under monitoring), and B.1.427/B.1.429 and B.1.616 (de-escalated variants) have been reported.

**Figure 1:**
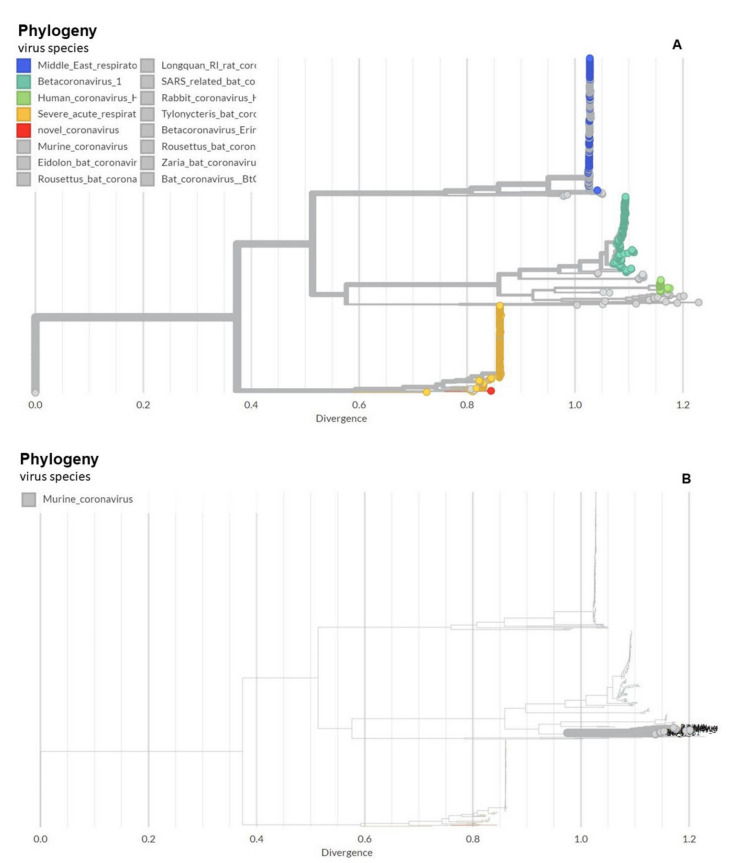
Phylogeny of coronaviruses including the novel coronavirus SARS-CoV-2. (**A**) Phylogeny of coronaviruses including the novel coronavirus SARS-CoV-2.; (**B**) Phylogeny of murine coronaviruses including the murine hepatitis virus.

Over the past 1000 years, coronaviruses have continually evolved [16]. The earliest identification of coronaviruses was in animals. It first isolated as an infectious bronchitis virus (IBV) in chickens in 1947 [17], followed by the isolation of mouse hepatitis virus (MHV) in mice in 1949 [18]. In 1946, pigs in the United States were identified as carriers of a transmissible gastroenteritis virus (TGEV) [18]. In the 1960s, human coronaviruses were isolated from respiratory tract infections [19]. B814 and 229E were the first two viruses isolated in humans [20,21]. Subsequently, tissue cultures were used to isolate additional coronavirus strains (OC16 and OC43) from humans [22,23]. To date, coronaviruses have been identified in numerous other species, including calves, dogs, cats, bats, sparrows, rabbits, and turkeys [24]. Early phylogenetic studies on SARS-CoV-2 genomic sequences showed that it clustered closely with sequences originating from SARS-like viruses from bats within lineage B of the betacoronavirus genus. Lineage A groups are prototypical coronaviruses such as MHV and the human coronaviruses HCoV-HKU1 and HCoV-OC43 (Figure 1A,B) ([25,26,27,28,29,30,31,32,33,34,35,36,37,38,39,40] tree.bio.ed.ac.uk/software/figtree/ accessed on 7 July 2021) (Software (ed.ac.uk accessed on 7 July 2021)), while the other highly pathogenic coronavirus, MERS-CoV, is found within lineage C, along with the related camel-derived MERS-CoV ([41,42] and references therein). The S protein amino acid sequences from four representative beta coronaviruses (HCoV-HKU1, MHV, SARS-CoV, and MERS-CoV) were aligned and the solved S protein structures were compared to determine their amino acid identity and the overall structural organization similarities among these proteins. The findings showed an average of approximately 30% identity among the four viral S proteins at the amino acid level, with the exception of HCoV-HKU1 and MHV, which share an amino acid identity of 59% at the S protein ([41,42] and references therein). While there are dissimilarities between the amino acid sequences, the structure of the four betacoronavirus S proteins were found to have similar folding patterns.

MHV-1 infection of A/J mice produces clinical SARS-like disease with high mortality [43,44,45]. These mice develop severe pulmonary disease at day 6 post-MHV-1 infection and 60% mortality from 7 to 12 days post-infection. On day 2, patchy interstitial alveolar thickening and fluid accumulation in alveolar spaces (pulmonary edema) are prominent. At death, the lungs show severe interstitial pneumonitis with large areas of complete consolidation. The interstitial inflammatory reaction includes the presence of hyaline membranes, fibrin deposition, and significant lymphocyte and macrophage infiltrates. Infiltrating cells in MHV-1-infected lung tissue from A/J mice are predominantly macrophages and neutrophils on days 2–6 post-MHV-1 infection. T cells (CD3 positive) also increase in the infiltrates, especially by day 6 post-infection. Examinations of the livers of MHV-1-infected A/J mice show normal histology at day 6, but on days 7 or 8 just prior to death, evidence of severe hepatic congestion has been observed, as in humans [46]. Thus, A/J mice are highly susceptible to MHV-1-induced pulmonary disease when the virus is delivered intranasally.

Since there are no preventive or therapeutic strategies to mitigate SARS-CoV-2 transmission and pathogenesis, and since MHV severe acute respiratory syndrome coronavirus (SARS-CoV), and SARS-CoV-2 share a common genus, the lessons learnt from MHV could offer mechanistic insights into SARS-CoV-2 infection in humans (ref [43], and references therein). While MHV-1 in mice and SARS-CoV-2 in humans share various similarities, there are also differences (e.g., the presence of spike protein binding receptors, ACE2 versus carcinoembryonic antigen-related cell adhesion molecule 1 (biliary glycoprotein) (CEACAM1a), also known as CD66a (cluster of differentiation 66a)) (ref [43], and references therein), as well as the proteolytic cleavage of four important amino acids at the S1/S2 site of the SARS-CoV-2 spike protein [47]. However, the similarities outweigh the differences [48,49], and our comprehensive histopathological findings, which are highly comparable to humans with COVID-19, strongly support the usefulness of the MHV-1 model for the study of SARS CoV-2 infection.

## 2. Materials and Methods

Female A/J mice (8 weeks of age, weighing 22 g) were purchased from Jackson Laboratories (Bar Harbor, ME, USA) and were maintained in micro-isolated cages (paired in a single cage), housed in the animal colony at the Biomedical Research Building animal isolation facility at the University of Miami Miller School of Medicine, Miami FL, USA, and fed a standard lab chow diet (Envigo 2918 irradiated, Teklad diet, Dublin, VA, USA) and water (autoclaved tap water) ad libitum. The study was conducted according to the guidelines of the University of Miami Institutional Animal Care and Use Committee (IACUC protocol number 20-131 LF) approved on 8 October 2020.

### 2.1. Viral Inocualtion and Experiental Group

MHV-1 was purchased from American Type Culture Collection (ATCC, cat# VR-261, Manassas, VA, USA). Mice were inoculated with 5000 PFU intranasally [44,45]. Briefly, 5 × 10^3^ PFU MHV-1 was mixed with 50 μL of ice-cold Dulbecco’s modified Eagle’s medium (DMEM, Gibco Cat# 11965-092, Lot# 2186816, ThermoFisher Scientific, Waltham, MA, USA) and instilled into the nares immediately, and mice were observed until the virus was inhaled. Experimental group: These mice were divided into 3 groups: (1) healthy control (*n* = 7); (2) infusion of healthy control with DMEM (used for intranasal infusion of MHV-1) (*n* = 5); and (3) MHV-1 alone (*n* = 16).

### 2.2. Clinical Observation

Mice challenged with MHV-1 were monitored for clinical signs. Clinical signs were scored by stages of (0) no clinical signs, (I) drowsiness and lack of movement, (II) slightly ruffled fur and altered hind limb posture, (III) ruffled fur and mildly labored breathing, (IV) ruffled fur, inactivity, moderately labored breathing, (V) ruffled fur, labored breathing and lethargy, and (VI) moribund state and death.

Mice with a disease stage of V–VI (occurring at 7–11 days) were weighed and euthanized, and their major organs, including lungs, liver, kidney, heart, and brain were removed and fixed in 10% formalin, processed routinely for paraffin sections, and stained with hematoxylin and eosin. Briefly, after gradient dehydration with various concentrations of alcohol in an automatic tissue dehydrator (HistoCore PELORIS 3 Premium Tissue Processing System, Leica Biosystems Inc., Buffalo Grove, IL, USA), tissues were embedded in paraffin blocks using a paraffin embedding station (HistoCore Arcadia Embedding Center, Leica Biosystems Inc., Buffalo Grove, IL, USA). The tissues were then cut into 10 μm thin slices by an ultra-thin semiautomatic microtome (Histocore autocut automated rotary microtome, Leica Biosystems Inc., Buffalo Grove, IL, USA) and adhered to the slides. After the slides were stained with hematoxylin and eosin (H&E), morphological changes were evaluated with a microscope (Olympus VS120 Automated Slide Scanner, Olympus, Pittsburgh, PA, USA) by a pathologist unaware of the treatment protocol.

To ascertain the extent of liver failure, blood was collected via cardiac puncture, and serum was used to measure liver enzymes. Levels of aspartate aminotransferase (AST), alanine aminotransferase (ALT), alkaline phosphatase (ALP), and bilirubin were determined at the onset of disease stages of V–VI, as previously described, using a Cobes 0501 automatic analyzer (Roche Diagnostics, Indianapolis, IN, USA) [50,51]. Body weight was measured day by day.

### 2.3. Statistical Analysis

Data were subjected to analysis of variance followed by Tukey’s multiple comparison test. A statistical analysis showing *p* < 0.05 was considered significant.

## 3. Results

### 3.1. Animal Survival Post-MHV-1 Infection

**Figure 2:**
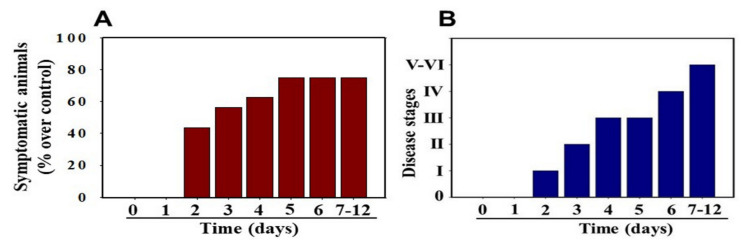
MHV-1 inoculated mice displayed signs of sickness. (**A**) About 40% of mice showed signs of sickness on day 2 post-MHV-1 inoculation, and the number of mice exhibiting clinical signs increased gradually (up to 75%). The remaining 25% of mice did not show any clinical signs (observed for up to 91 days). (**B**) MHV-1 inoculated mice showed clinical signs (from stages I to VI) from days 2 to 12 and beyond. Mild to moderate clinical signs (stages I–III) were observed from days 2 to 4. The MHV-1 infected mice showed severe sickness (stages IV–VI) from day 6 onward. Further, 60% of animals died from days 7 to 12 (*n* = 16).

MHV-1 inoculated female mice displayed signs of sickness (drowsiness and lack of movement) at 2 days post-exposure. About 43.75% of mice (7/16) showed signs of illness on day 2 post-MHV-1 inoculation, and the number of mice exhibiting clinical signs increased gradually (56.25%, 62.5%, and 75.0% on days 3, 4, and 5, respectively) (Figure 2A). Of note, the remaining 25% of mice did not show any clinical signs (observed for up to 91 days). Further, exposure of mice to DMEM alone had no effect on animal survival.

**Figure 3:**
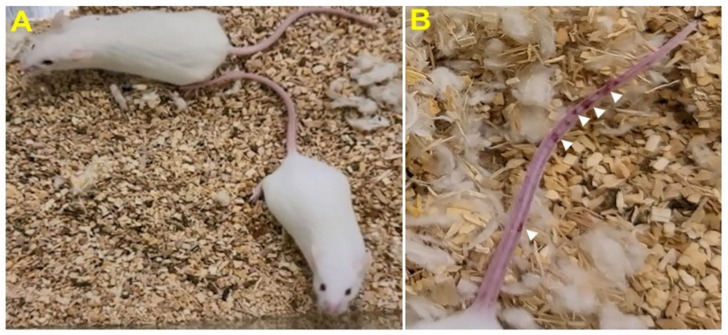
MHV-1 inoculated mice displayed severe disease. (**A**) Normal mouse. (**B**) A representative mouse from the MHV-1 inoculated group showing venous thrombosis consistent with symptoms in patients with COVID-19 (*n* = 11).

Stage I symptoms (drowsy and lack of movement) on day 2 and stage II symptoms (slightly ruffled fur and altered hind limb posture) on day 3 were observed post-MHV-1 inoculation. Stage III symptoms (ruffled fur and mildly labored breathing) were detected on days 4 and 5. Stage IV (ruffled fur, inactivity, moderately labored breathing, and tremor) was observed on day 6, and stages V and VI (ruffled fur, obvious labored breathing and lethargy, as well as a moribund state and death) were noted from days 7 to 12 (Figure 2B). Figure 2A,B involves the same animals. For example, in Figure 2A, approximately 40% of animals showed clinical signs of infection and all of them were in stage I. On day 3, about 55–60% animals showed clinical signs of infection that were of stage II. The additional mice (10–20%) that exhibited clinical signs of infection at later stages showed a severity similar to that of mice that exhibited clinical signs of infection at early stages. Of note, we observed mild diarrhea (several loose stools in one day) in the early stages of illness (from 2 to 4 days). However, the diarrhea ended from days 5 to 12 and occurred only in a few animals (5/16 mice), as observed in humans in association with COVID-19 [52,53]. Further, MHV-1 inoculated mice displayed severe venous thrombosis (4/16 mice at days 5–7), a characteristic feature of COVID-19 (Figure 3) [38,39,40,41,42,54,55,56,57,58]. Milder forms of thrombosis were also observed in MHV-1 inoculated mice at days 5–7 (7/16). The natural history of the disease was observed in mice that survived after day 12 for up to 91 days.

### 3.2. Weight Loss Post-MHV-1 Infection

**Figure 4:**
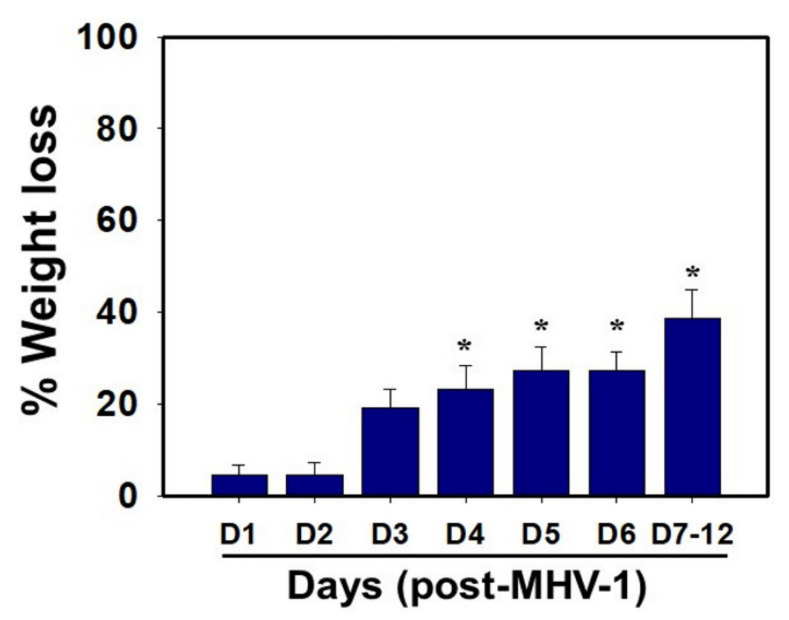
Body weight losses in MHV-1 infected mice. MHV-1 inoculated mice lost 20–40% of body weight over days 3–8, corresponding well with the severity of the disease (*n* = 16). * *p* < 0.05 versus control.

MHV-1 inoculated mice were monitored for weight loss from day 2 onwards. MHV-1 administered mice showed approximately 20% weight loss on day 3, around 25–30% weight loss on day 4 and 5, and 35–40% weight loss from days 7 to 12 (Figure 4). Of note, the weight loss occurred rapidly on days 6–7 in animals that showed severe clinical signs (from about 25% to 40%). We were unable to control this since it was not obvious whether any particular animal would lose that much weight in a short period of time, and as such we could not provide glucose/KCl (i.e., fluid support). The reason for such rapid weight loss is unknown. It is possible that rapid body fluid loss or a lack of fluid intake and loss of muscle mass and fat during the end stages may have occurred, as has frequently been observed in humans in association with SARS-CoV-2 infection. While our elaborated survival/characterization study shows the exact nature of the viral infection, and our initial characterization study will assist researchers in better understanding the severity of disease progression and in designing mechanistic studies, we will include the humane endpoint criteria in future investigations based on our current observations.

We examined the status of liver enzymes. Increased liver enzymes (ALT and AST) were identified in MHV-1-infected mice, as observed in patients with drug or chemical-induced acute liver failure or in association with COVID-19. AST levels increased manifold in MHV-1 inoculated mice as compared to un-inoculated mice (3459.2 ± 684.1 units/L in MHV-1 infected mice as compared to 96.8 ± 14.2 units/L in the control group, 34.7-fold increase over control). There was also an increase in ALT levels (3068.5 ± 861.3 units/L in MHV-1 infected mice as compared to 31.5 ± 11.6 in the control group, 96.4-fold increase over control). ALP and bilirubin levels were also increased in infected mice (986.3 ± 178.4 units/L of ALP in MHV-1 infected mice as compared to 589.1 ± 108.7 in the control group, 67% increase over control; and 0.86 ± 0.2 mg/L of bilirubin, as compared to 0.075 ± 0.02 mg/L in uninfected mice, 10.4-fold increase over control) (Table 1). Furthermore, the exposure of mice to DMEM had no effect on alterations in liver enzymes (i.e., levels were identical to those of healthy controls). These findings strongly suggest that MHV-1 leads to severe liver injury; similar observations have been made in patients with SARS-CoV-2 infection [46,59,60,61,62,63].

### 3.3. Histopathological Changes Post-MHV-1 Infection

**Figure 5:**
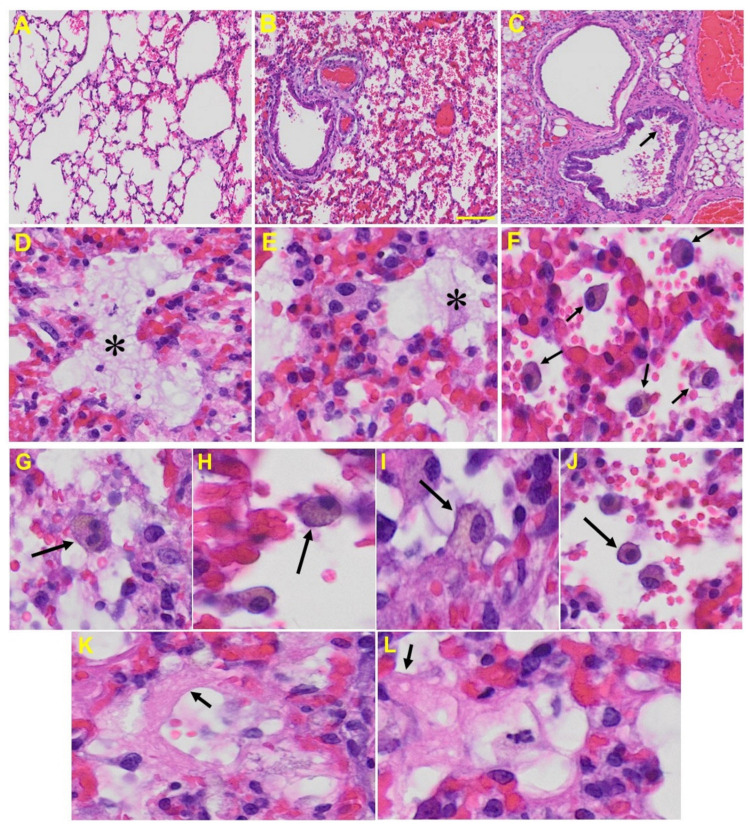
Lung from MHV-1 exposed mice. (**A**) Normal mouse. (**B**) MHV-1-infected mouse lung at day 7. (**C**) The MHV-1 infected mouse lung shows inflammation, granular degeneration of cells, and migration of leukocytes into the lungs (arrow). (**D**,**E**) Proteinaceous debris filling of the alveolar spaces with fibrillar to granular eosinophilic protein strands caused by progressive breakdown of the capillary wall and epithelial integrity, permitting the leakage of protein-rich edema fluid into the alveoli (asterisk) (commonly seen in ARDS). (**F**–**J**) Presence of hemosiderin-laden macrophages, indicating pulmonary congestion with dilated capillaries and leakage of blood into alveolar spaces. Further, peribronchiolar interstitial infiltration, bronchiole epithelial cell necrosis, necrotic cell debris within alveolar lumens, alveolar exudation, infiltration, hyaline membrane formation (**K**,**L**), and alveolar hemorrhage with red blood cells within the alveolar space and interstitial edema were also observed in these mice (H&E original magnification 400× Figure 5A–C; scale bar image divided by actual scale bar length).

*Lung:* MHV-1-infected mice at day 7 showed inflammation (i.e., granular degeneration of cells, and migration of leukocytes into the lungs), along with proteinaceous debris filling of the alveolar spaces with fibrillar to granular eosinophilic protein strands caused by the progressive breakdown of the capillary wall and epithelial integrity, permitting leakage of protein rich edematous fluid into the alveoli, and the presence of hemosiderin-laden macrophages (indicating pulmonary congestion with dilated capillaries and leakage of blood into alveolar spaces). Furthermore, peribronchiolar interstitial infiltration, bronchiole epithelial cell necrosis, necrotic cell debris within alveolar lumens, alveolar exudation, hyaline membrane formation, alveolar hemorrhage with red blood cells within the alveolar space, and interstitial edema are all characteristic features of infected lungs in humans with SARS-CoV-2 infection [64] (Figure 5).

**Figure 6:**
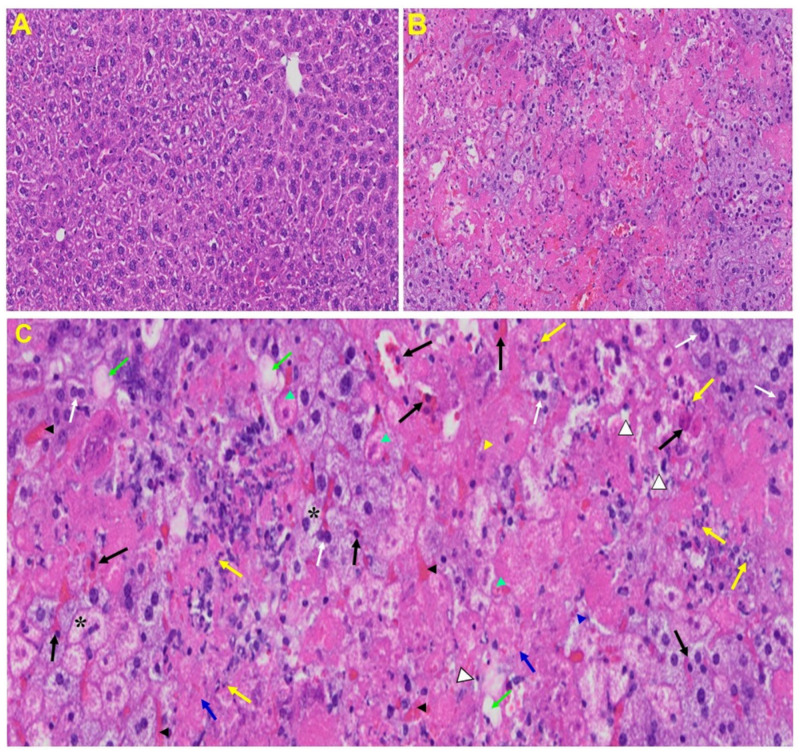
Liver from MHV-1 exposed mice. (**A**) Normal mouse. (**B**) Infected mouse liver at day 7. (**C**) The MHV-1 infected mouse liver at day 7 shows hepatocyte degeneration (yellow arrows), severe periportal hepatocellular necrosis with pyknotic nuclei (black arrows), severe hepatic congestion (black arrowheads), ballooned hepatocytes (asterisks), vacuolation (green arrows), and the presence of piecemeal necrosis (blue arrowhead), as well as hemorrhagic changes. Ground glass hepatocytes show voluminous, abundant, granular cytoplasm, peripheral cytoplasmic clearing and central nuclei, and apoptotic (acidophil) bodies. Several hepatocytes are absent and have been replaced by abundant inflammatory cells (yellow arrowhead). Note the condensation and dark staining of the cytoplasm and the absence of the nucleus (green arrowheads), fatty changes (blue arrows), binucleated hepatocytes (white arrows), and activated Kupffer cells (white arrowheads), indistinctive of severe liver failure (H&E original magnification of A&B is 400× as noted in Figure 5B).

*Liver*: Liver from MHV-1 exposed mice showed hepatocyte degeneration, severe periportal hepatocellular necrosis with pyknotic nuclei, severe hepatic congestion, ballooned hepatocytes, vacuolation, and the presence of piecemeal necrosis, as well as hemorrhagic changes. Ground glass hepatocytes showed voluminous, abundant, granular cytoplasm, peripheral cytoplasmic clearing and central nuclei, and apoptotic (acidophil) bodies, as well as absent hepatocytes replaced by abundant inflammatory cells. Condensation and dark staining of the cytoplasm, an absence of the nucleus, fatty changes, binucleated hepatocytes, and activated Kupffer cells were also observed in MHV-1 exposed mice livers (Figure 6).

**Figure 7:**
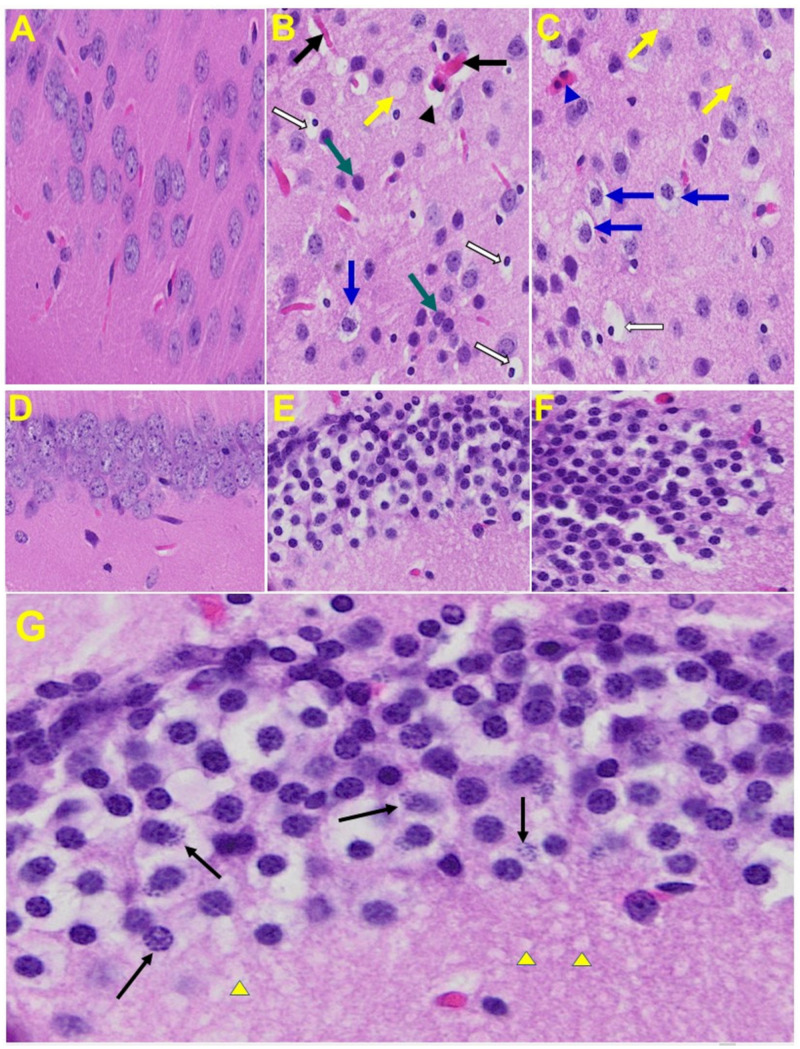
MHV-1 infected mouse brain. (**A**) Normal mouse. (**B**,**C**) The infected mouse brain cortex at day 7 shows congested blood vessels (black arrows), perivascular cavitation (black arrowhead) suggestive of edema, pericellular halos (blue arrows), vacuolation of neuropils (yellow arrow), darkly stained nuclei (curved arrows green arrows), pyknotic nuclei amid associated vacuolation of the neuropil (white arrows), and acute eosinophilic necrosis (blue arrowhead). (**D**) Normal mice. (**E**,**F**) The brain hippocampus of an MHV-1 infected mouse (enlarged image, (**G**)) shows a necrotic neuron with fragmented nucleus (arrow) and vacuolation (arrowhead) (H&E original magnification of A–F is 400× as noted in Figure 5B).

*Brain*: Upon examination of the MHV-1 infected mice brain (Figure 7B,C), we observed congested blood vessels, perivascular cavitation (suggestive of edema), pericellular halos, vacuolation of neuropils, darkly stained nuclei and pyknotic nuclei amid associated vacuolation of the neuropil, and acute eosinophilic necrosis. The brain hippocampus of MHV-1 infected mice showed necrotic neurons with fragmented nuclei and vacuolation (Figure 7).

**Figure 8:**
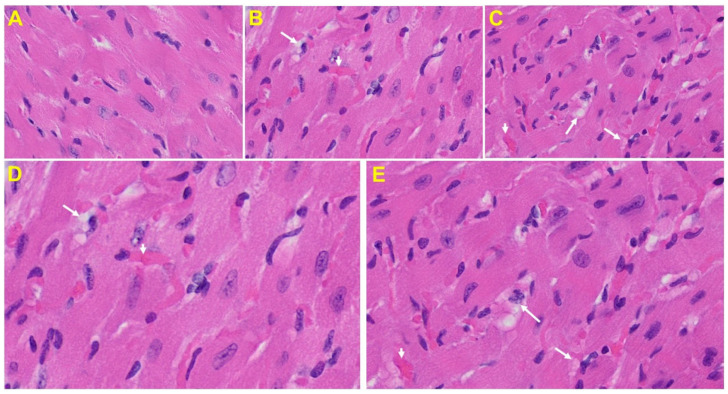
An MHV-1 infected mouse heart. (**A**) Normal mouse heart. (**B**,**C**) MHV-1 infected mouse heart showing severe interstitial edema (long arrows), vascular congestion and dilation (short arrows), and red blood cell extravasation into the interstitium (enlarged images of an MHV-1 infected mouse heart (**D**,**E**)) (H&E original magnification of A–C is 400× in A–C as noted in Figure 5B).

*Heart*: The heart of MHV-1 infected mice showed severe interstitial edema, vascular congestion and dilation, and red blood cells infiltrating between degenerative myocardial fibers (Figure 8).

**Figure 9:**
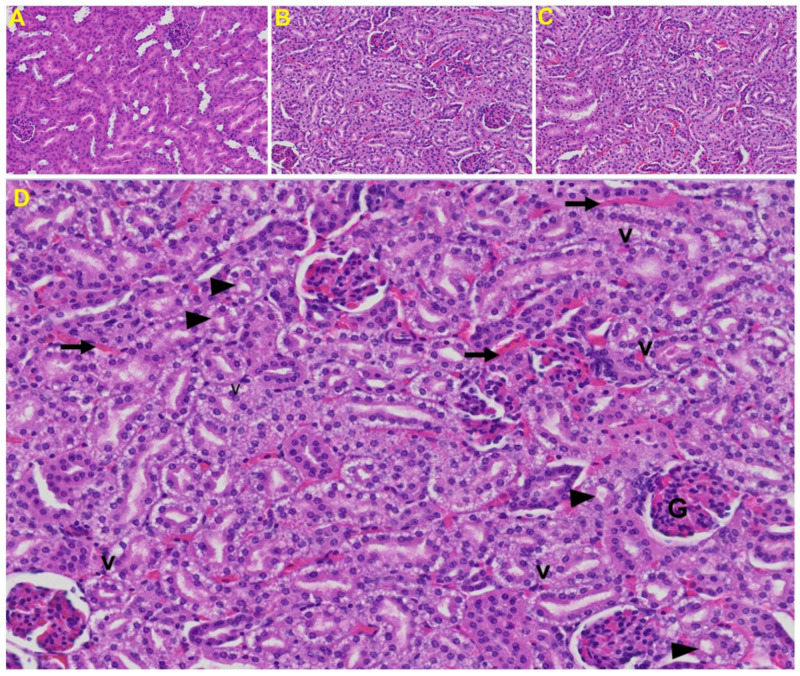
Kidney from an MHV-1 infected mouse. (**A**) Normal mouse kidney. (**B**,**C**) Kidney sections from an MHV-1 infected mouse. Kidney sections from a control mouse showing a normal histological structure with the glomerulus, proximal convoluted tubule, and distal convoluted tubules. (**D**) Enlarged image of a kidney from an MHV-1 infected mouse showing proximal and distal tubular necrosis (arrowhead), hemorrhage in the interstitial tissue (long arrow), and vacuolation of renal tubules (V) (H&E original magnification of A–C is 400× as noted in Figure 5B).

*Kidney*: Tubular epithelial cell degenerative changes, peritubular vessel congestion, proximal and distal tubular necrosis, hemorrhage in interstitial tissue, and vacuolation of renal tubules were observed in MHV-1 exposed mice kidneys (V) (Figure 9).

## 4. Discussion

Our study demonstrates MHV-1-induced histopathological changes in various organs, as well as weight loss, in addition to animal death. MHV-1-infected mice showed severe lung inflammation, granular degeneration of cells, and migration of leukocytes into the lungs. Furthermore, peribronchiolar interstitial infiltration, bronchiole epithelial cell necrosis and necrotic cell debris within alveolar lumens, alveolar exudation, infiltration, hyaline membrane formation, and alveolar hemorrhage with red blood cells within the alveolar space and interstitial edema were also observed in these mice. When compared to uninfected mice, those infected showed liver hepatocyte degeneration, severe cell necrosis, and hemorrhagic changes. Tubular epithelial cell degenerative changes and vacuolation, as well as peritubular vessel congestion, were observed in kidneys. The heart of MHV-1 infected mice showed severe interstitial edema, vascular congestion and dilation, and red blood cells infiltrating between degenerative myocardial fibers. We also observed vacuolation of the brain cortex, congested blood vessels within perivascular spaces, pyknotic nuclei and associated vacuolation of the neuropil in the cortex, and acute eosinophilic necrosis and necrotic neurons with fragmented nuclei and vacuolation in the hippocampus. Our findings suggest that the widespread vascular and cellular events seen in the highly relevant surrogate animal model represent an attractive and safe model for the study of SARS-CoV-2 infection, pathophysiologic mechanisms, and potential therapeutic interventions.

Laboratory MHV strains have been broadly studied to identify harmful coronavirus factors and elucidate host mechanisms of antiviral pathogenicity (see, [65,66,67,68] and references therein). The major MHV viruses used are MHV-3, MHV-A59, MHV-JHM, and MHV-S. Animals susceptible to MHV-3 generate an early increased proinflammatory response and a predominant Th2 cytokine profile leading to the activation of coagulation and tissue necrosis, whereas resistant animals generate a predominant TH1 immune response leading to the production of cytotoxic T-lymphocytes and protective B-cell responses [69,70,71], but not CD8 T-cell response [72]. Furthermore, the macrophage prothrombinase FGL2/fibroleukin is an important determinant of disease in MHV-3 induced fulminant hepatitis, and the coronavirus nucleocapsid gene mediates much of its effect by inducing FGL2/fibroleukin [73,74,75]. However, MHV-A59 and MHV-3 infected mice all developed severe hepatic necrosis and died of liver failure by day 10, with pulmonary lesions that were comparatively less severe as compared to those generated by MHV-1, and did not have the characteristics of lesions described in patients with SARS-CoV-1 and -2 infection. A recent study also examined whether senescent cells are a cause of adverse outcomes of infection with aging in MHV-A59 infected mice, thus providing a potential treatment strategy to alleviate COVID-19 through the amelioration of inflammation [76].

In contrast to MHV-A59-and MHV-3, BALB/cJ mice inoculated intranasally with MHV-S and MHV-JHM did not develop liver or lung disease [44]. Conversely, MHV-1 infection of A/J mice produces clinical and pathological SARS-like disease with high mortality, even when compared with MHV-1 inoculated C57BL/6J and C3H/St strains, which exhibit relative resistance and have an intermediate susceptibility, respectively, or clear the virus efficiently and survive [28]. Moreover, marked elevations in IL-6, IL-10, IFN-γ, TNF-α, and CCL2 expression were observed in MHV-1 infected mice in addition to severe immune reactions as compared to other mouse strains exposed to MHV-A59, MHV-3, MHV-S, or MHV-JHM. Additionally, we showed significant pathological changes in various organs that are closely comparable to those observed in humans in association with SARS CoV-2 infection [44].

More recently, mice sensitized with human ACE2 have been considered as a relevant model for the study of SARS-CoV-2 infection. While mice sensitized with Ad5-hACE2 or AAV-hACE2 are useful for evaluating vaccines and antiviral therapies as well as for identifying SARS-CoV-2 specific antibody/immune cell epitopes, a limitation with these mice and in some of the transgenic mice expressing human ACE2 is that human ACE2 is expressed ectopically, which changes the tissue or cellular tropism of the virus [43,77]. There are also other models including SARS-CoV-2 exposure in Syrian hamsters, mink, ferrets, cats, dogs, and pigs. These animal models display limitations (including moderate respiratory signs (mink)), and replication appears to be restricted to the respiratory and gastrointestinal tracts (in ferrets), although the ACE2 sequence of humans known to interact with the receptor-binding domain of the SARS-CoV-2 spike glycoprotein is present in these animals [77]. Thus, our findings in the MHV-1 mouse model, along with earlier observations, provide considerable insights into the potential pathogenic factors that may be involved in SARS CoV-2 infection.

The most common clinical presentation of severe COVID-19 is acute respiratory failure consistent with the acute respiratory distress syndrome (ARDS). Studies have shown diffuse alveolar damage with hyaline membrane formation, pneumocyte activation, microvascular thrombi, lymphocytic inflammation, proteinaceous edema, vascular remodeling via intussusceptive angiogenesis in the presence of microvascular thrombi, fibrosis, chronic inflammation, loose fibrous plugs associated with organizing pneumonia, endothelial injury with vacuolization of the cytoplasm and detachment of cells in small and medium-sized pulmonary arteries, deposition of fibrin and erythrocytes in the alveolar spaces and septa, hemorrhage, and hemosiderin deposition accompanied by complement complex deposition (especially near the alveolar capillaries), as well as alveolar type II (AT2) cell hyperplasia, fibrin exudates, vascular congestion, and mononuclear and multinucleated giant cell alveolar inflammation (with a noted absence of neutrophilic inflammation) in humans with COVID-19 [64,78,79,80,81,82].

It should be highlighted that the above-mentioned changes were recapitulated in MHV-1 infection in mice. More precisely, proteinaceous debris filling the alveolar spaces with fibrillar to granular eosinophilic protein strands was observed. This may have been caused by progressive breakdown of capillary walls and epithelial integrity, which permits the leakage of protein rich edema fluid into the alveoli—events commonly seen in ARDS [83]. This proteinaceous fluid presents itself as a lightly eosinophilic material that ranges from homogenous to fibrillar (i.e., fibrin strands). Furthermore, the presence of hemosiderin-laden macrophages, an iron storage complex that is composed of partially digested ferritin and lysosomes, was seen frequently, indicative of pulmonary congestion with dilated capillaries and leakage of blood into alveolar spaces. The breakdown of heme gives rise to biliverdin and iron. The body then traps the released iron and stores it as hemosiderin in tissues. Hemosiderin is also generated from the abnormal metabolic pathway of ferritin [84]. Our findings collectively suggest major defects in the lung of MHV-1 infected mice, similar to those observed in humans in association with SARS-CoV-2 infection.

Mild and transient liver injury, as well as severe liver damage, can occur in COVID-19 patients. Wong et al. [85] indicated that 14.8–53.1% of COVID-19 patients had abnormal levels of alanine aminotransferase, aspartate aminotransferase, and bilirubin during the disease, in accordance with the observations made in the current study. Furthermore, these authors reported that the severity of liver damage is proportional to that of COVID-19. The level of alkaline phosphatase (ALP) was also increased in MHV-1 inoculated mice. However, the levels of bilirubin and ALP were lower than those observed with AST and ALT. Similar findings were observed in patients with COVID-19 [85], suggesting that the insult in SARS-CoV-2 infection may predominantly have been due to hepatocellular injury, as opposed to obstruction in bile flow or absence of alcohol or other secondary chemical injuries during SARS-CoV-2 infection.

Multi-organ failure is generally accompanied by short- and long-term neurological conditions [86,87]. It has been suggested that viral invasion of the central nervous system by SARS-CoV-2 is made possible by the synapse-connected route observed with other coronaviruses such as SARS-CoV, and can lead to several neurological complications including ataxia, seizures, neuralgia, unconsciousness, acute cerebrovascular disease and encephalopathy, anosmia, cognitive and attention deficits (i.e., brain fog), new-onset anxiety, depression, psychosis, and even suicidal behavior [71,72,73,74,88,89,90,91]. A follow-up study conducted in Germany and the United Kingdom found neuropsychiatric symptoms in about 20–70% of patients in association with SARS-CoV-2 infection even after the disappearance of respiratory symptoms [74], suggesting that brain dysfunction persists even after symptoms have resolved. Recently, Mao et al. [92] reported that 36.4% of their cohort with COVID-19 had neurologic manifestations, with the severe group being more likely to have acute cerebrovascular disease, impaired consciousness, and skeletal muscle injury.

Histopathologically, inflammatory cell cuffs around small blood vessels and degenerative neurons, inflammatory cell infiltration and focal hemorrhages were observed in humans with COVID-19 [80]. These findings correlate well with our observations in MHV-1 infected mice, strongly suggesting the usefulness of the mice model to study mechanisms occurring in SARS-CoV-2 infection in humans.

Nearly one-fourth of those hospitalized with COVID-19 are diagnosed with cardiovascular complications, which have been shown to contribute to approximately 40% of all COVID-19-related deaths. A recent study used cardiac MRIs on 100 individuals who had recovered from COVID-19 within the past 2 to 3 months, finding abnormalities in the heart of 78% of recovered patients and “ongoing myocardial inflammation” in 60% [93]. The same study found high levels of the blood enzyme troponin, an indicator of heart damage, in 76% of patients tested, even though heart function appeared to be generally preserved. Furthermore, current evidence demonstrates myocardial inflammation with or without direct cardiomyocyte damage in humans with SARS-CoV-2 infection [77,78,79,80,94,95,96,97]. Myocarditis results from direct heart invasion by the virus itself or more commonly by inflammation caused by cytokine storm, resulting in an enlarged and weakened heart, leading to low blood pressure and fluid deposition in the lungs. Histopathological evidence suggests the presence of hypertrophied cardiomyocytes along with inflammatory infiltrates, focal edema, interstitial hyperplasia, fibrosis, degeneration, necrosis and signs of lymphocytic myocarditis, the presence of CD4 T-cells along with other inflammatory infiltrates in myocardium, endocarditis and inflammation of interstitial tissue, damaged cell membrane interstitial cells, and leukocyte infiltration in humans with SARS-CoV-2 infection [48,64,81,82,83,84,98,99,100,101]. We also identified many events in MHV-1 infected mouse hearts that have been observed in humans with SARS-CoV-2 infection. These include severe interstitial edema, vascular congestion and dilation, and red blood cells infiltrating between degenerative myocardial fibers.

Acute kidney injury (AKI) is an abrupt loss of kidney function which develops within 7 days in patients with SARS and MERS-CoV. Early reports indicate that up to 30% of patients hospitalized with COVID-19 in China and the USA developed moderate to severe kidney injury. Kidney disease among patients with COVID-19 can manifest as AKI, hematuria, or proteinuria, and portends a higher risk of mortality. As in other organs, it remains unclear if AKI is largely due to hemodynamic changes and cytokine release or if the virus also leads to direct cytotoxicity. Kidney histopathology examined in an autopsy series of 42 patients who died with COVID-19 showed varying degrees of acute tubular necrosis, collapsing focal segmental glomerulosclerosis [85,86,87,88,89,90,91,102,103,104,105,106,107,108], interstitial infiltration by lymphocytes, tubular epithelial cell necrosis, fibrinoid necrosis of blood vessels, and microthrombi in small vessels. There were also erythrocyte casts in some of the tubules (erythrocyturia), as well as ballooned glomeruli with mild mesangial expansion akin to diabetic nephropathy class IIa [80,109,110,111,112,113,114,115,116]. We also found similar events when mice were inoculated with MHV-1. These include proximal and distal tubular necrosis, hemorrhage in interstitial tissue, and vacuolation of renal tubules.

## 5. Conclusions

In conclusion, earlier reports and our current findings in this model demonstrate immune and inflammatory responses. These include lung inflammation, peribronchiolar interstitial infiltration, bronchiolar epithelial cell necrosis, intra-alveolar necrotic debris, alveolar mononuclear cell infiltration, hyaline membrane formation, the presence of hemosiderin-laden macrophages, interstitial edema, severe liver vascular congestion, luminal thrombosis of portal and sinusoidal vessels, hepatocyte degeneration, cell necrosis and hemorrhagic changes, proximal and distal tubular necrosis, hemorrhage in interstitial tissue, vacuolation of renal tubules, severe interstitial edema, vascular congestion and dilation along with red blood cell extravasation into the interstitium, congested blood vessels, perivascular cavitation, cortical pericellular halos, vacuolation of neuropils, darkly stained nuclei, pyknotic nuclei and associated vacuolation of the neuropil in the cortex, acute eosinophilic necrosis, necrotic neurons with fragmented nuclei, and vacuolation in the hippocampus. It should be highlighted that all these events strongly suggest widespread thrombotic events, mimicking the reported findings in SARS-CoV-2 infected humans and thus indicating an attractive, safe animal model for the study of SARS-CoV-2 infection, pathophysiologic mechanisms, and potential therapeutic interventions.

## Figures and Tables

**Table 1:** Liver enzymes of mice exposed to MHV-1.

	Uninfected Mice	MHV-1 Infected Mice
AST (Units/L) ALT (Units/L) ALP (Units/L)	96.8 ± 14.2 31.5 ± 11.6 589.1 ± 108.7	3459.2 ± 684.1 * 3068.5 ± 861.3 * 986.3 ± 158.4 *
Bilirubin (mg/L)	0.075 ± 0.02	0.86 ± 0.2 *

Mean values ± SD. *statistically significant difference from uninfected mice. AST, Aspartate aminotransferase; ALT, Alanine aminotransferase; ALP, alkaline phosphatase.

## Data Availability

The data presented in this study are available on request from the corresponding author. The data are not publicly available due to the University of Miami Miller School of Medicine’s privacy policy.
